# Fungal plant cell wall-degrading enzyme database: a platform for comparative and evolutionary genomics in fungi and Oomycetes

**DOI:** 10.1186/1471-2164-14-S5-S7

**Published:** 2013-10-16

**Authors:** Jaeyoung Choi, Ki-Tae Kim, Jongbum Jeon, Yong-Hwan Lee

**Affiliations:** 1 Fungal Bioinformatics Laboratory, Seoul National University, Seoul 151-921, Korea; 2 Department of Agricultural Biotechnology, Seoul National University, Seoul 151-921, Korea; 3 Center for Fungal Pathogenesis, Seoul National University, Seoul 151-921, Korea; 4 Center for Fungal Genetic Resource, Seoul National University, Seoul 151-921, Korea; 5 Plant Genomics and Breeding Institute, Seoul National University, Seoul 151-921, Korea; 6 Research Institute for Agriculture and Life Sciences, Seoul National University, Seoul 151-921, Korea

## Abstract

**Background:**

Plant cell wall-degrading enzymes (PCWDEs) play significant roles throughout the fungal life including acquisition of nutrients and decomposition of plant cell walls. In addition, many of PCWDEs are also utilized by biofuel and pulp industries. In order to develop a comparative genomics platform focused in fungal PCWDEs and provide a resource for evolutionary studies, Fungal PCWDE Database (FPDB) is constructed (http://pcwde.riceblast.snu.ac.kr/).

**Results:**

In order to archive fungal PCWDEs, 22 sequence profiles were constructed and searched on 328 genomes of fungi, Oomycetes, plants and animals. A total of 6,682 putative genes encoding PCWDEs were predicted, showing differential distribution by their life styles, host ranges and taxonomy. Genes known to be involved in fungal pathogenicity, including polygalacturonase (PG) and pectin lyase, were enriched in plant pathogens. Furthermore, crop pathogens had more PCWDEs than those of rot fungi, implying that the PCWDEs analysed in this study are more needed for invading plant hosts than wood-decaying processes. Evolutionary analysis of PGs in 34 selected genomes revealed that gene duplication and loss events were mainly driven by taxonomic divergence and partly contributed by those events in species-level, especially in plant pathogens.

**Conclusions:**

The FPDB would provide a fungi-specialized genomics platform, a resource for evolutionary studies of PCWDE gene families and extended analysis option by implementing Favorite, which is a data exchange and analysis hub built in Comparative Fungal Genomics Platform (CFGP 2.0; http://cfgp.snu.ac.kr/).

## Background

Plant cell wall-degrading enzymes (PCWDEs) play significant roles throughout the fungal life including acquisition of nutrients and decomposition of plant cell walls. Particularly for plant pathogens, it is critical to decide where and when to start intruding into the host cell. Many plant pathogens are known to secrete a variety of PCWDEs to perceive weak regions of plant epidermal cells and penetrate the plant primary cell wall. For example, a cutinase (CUT2) in the rice blast fungus, *Magnaporhte oryzae*, is known to play roles in hydrophobic surface sensing, differentiation and virulence on rice and barley [1]. As another example of cutinase, disruption of CutA from *Fusarium solani *f. sp. *pisi *is responsible for decreased virulence on pea [2]. Additionally, degradation of xylan and pectin is required for fungal pathogens to invasively penetrate and proliferate inside host cells. In *M. oryzae*, some endoxylanases are thought to be responsible for fungal pathogenicity, even if three of them, XYL1, XYL2 and XYL6, are not required for pathogenicity [3]. According to the analysis between life styles and eight substrates including xylan and xyloglucan, pathogenic fungi showed more hydrolytic activities [4] implying the importance of these enzymes. Among the pectinolytic enzymes, many characterized polygalacturonases (PGs), Bcpg1, Cppg1-2 and P2c from *Botrytis cinerea*, *Claviceps purpurea *and *Aspergillus flavus*, respectively, are known to be responsible for successful infection on their hosts [5-7]. Besides the phytopathological impact mentioned above, PCWDEs have attained a lot of attention for their potential applications in pulp and biofuel industries, to find and develop the most economic and efficient combinations of enzymes to yield fermentable saccharides from plant biomass [4].

**Figure 1:**
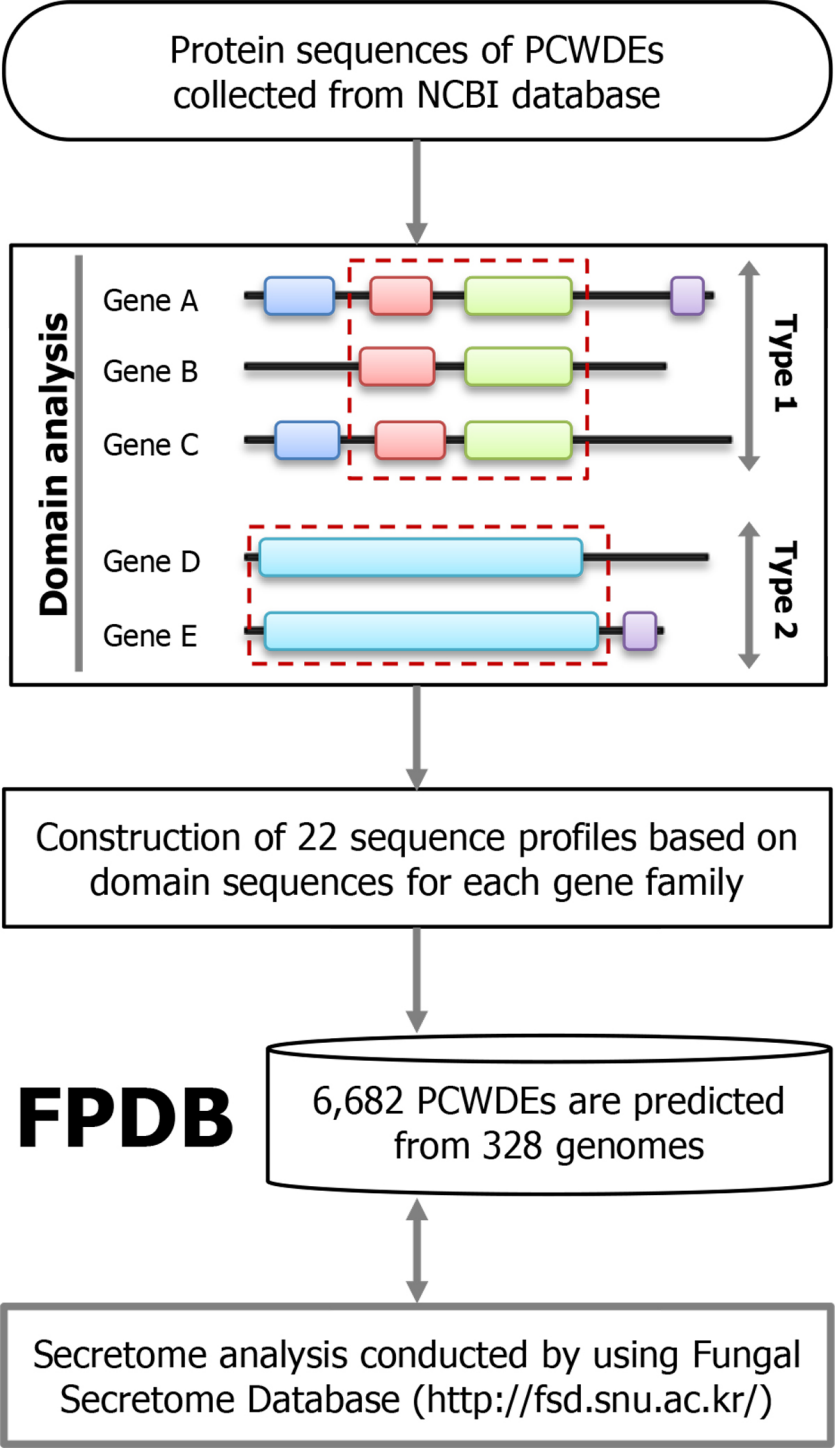
**A constructed pipeline for prediction of PCWDEs**. *In silico *prediction pipeline in the FPDB is illustrated as a flowchart. See Materials and Methods section for more details of each process.

Even though a large number of genomes are available, there is no systematic platform for dissecting the genes encoding PCWDEs especially in the fungal kingdom. Although Carbohydrate-Active Enzymes (CAZY) database archives a wide spectrum of glycosyl hydrolases [8], it is not focused on fungi and not all of them are PCWDEs. In order to understand fungal PCWDEs in kingdom level, we developed a new web-based platform, Fungal PCWDE Database (FPDB; http://pcwde.riceblast.snu.ac.kr/), to identify and classify genes encoding PCWDEs from fungal genomes (Figure 1).

We selected four major components of plant cell wall that are well-studied and/or critical for pathogen-host interactions. Subsequently, 22 gene families, including five subfamilies, are selected by materials they degrade (Table 1). First of all, cuticle layer is the outermost barrier of plant epidermal tissue and important for that it is the first defence line against pathogens. Another component is pectin which constructs major skeleton of plant cell walls and is hard to degrade. The others are cellulose and hemicellulose, the most plentiful components of the primary cell wall, including xylan, xyloglucan and galactoglucomannan [9,10]. The 22 gene families have been divided into two categories, main-chain degrading and accessary PCWDEs. The main-chain degrading PCWDEs participate in breakdown of highly polymeric backbone compounds, such as cutin polymer, (gluco)xylan, pectin or glucan. On the other hand, accessary PCWDEs degrade derivatives that the main-chain degrading PCWDEs produce, for example, xylobiose or many forms of oligo-/di-saccharides into respective monomers, hence producing ready-to-use carbon sources (Table 1).

**Table 1:** List of gene families archived in the FPDB

Substrate	Category	Gene Family	Number of Genes	Number of Genomes
Cutin	Leaf Surface	Cutinase	112	39

Cellulose	Main-chain degrading	Cellobiohydrolase (Type 1)	174	59
	Main-chain degrading	Cellobiohydrolase (Type 2)	71	35
	Accessary	Alpha-glucosidase (Type 1)	1,060	304
	Accessary	Alpha-glucosidase (Type 2)	834	197

Pectin	Main-chain degrading	Alpha-rhamnosidase	178	53
	Main-chain degrading	Pectate lyase	119	39
	Main-chain degrading	Pectin lyase	130	38
	Main-chain degrading	Polygalacturonase	713	163
	Main-chain degrading	Rhamnogalacturonan lyase	96	50
	Accessary	Beta-D-galactosidase (Type 1)	90	59
	Accessary	Beta-D-galactosidase (Type 2)	262	104
	Accessary	Endoarabinase	43	31
	Accessary	Pectin methylesterase	448	77
	Accessary	Rhamnogalacturonan acetylesterase	57	45

Xylan	Main-chain degrading	Endoxylanase (Type 1)	171	64
	Main-chain degrading	Endoxylanase (Type 2)	122	51
	Accessary	Alpha-glucuronidase	41	35

Galacto(gluco)mannan	Main-chain degrading	Alpha-mannosidase (Type 1)	1,310	300
	Main-chain degrading	Alpha-mannosidase (Type 2)	267	242
	Main-chain degrading	Beta-endo-mannnanase	176	67
	Main-chain degrading	Beta-mannosidase	208	147


In this study, we summarize the inventory of fungal genes encoding PCWDEs over the taxonomy. In addition, we also conduct comparative genomic analysis to elucidate differences among various fungal life styles and host ranges regarding the roles of PCWDEs in fungal pathogenesis. Lastly, evolutionary duplications and losses of genes encoding PGs are analyzed to elucidate more about the differential distribution of genes encoding PCWDEs.

## Results and discussion

### Identification of genes encoding PCWDEs

From 328 genomes, 6,682 genes are predicted to encode 22 gene families of PCWDEs (Figure 1). To evaluate the confidence level of the predicted genes, we performed the statistical analysis with positive and negative sets from UniProtKB/SwissProt [11], a manually curated protein database. The sensitivity and specificity reached to 95.31% and 98.55%, respectively. These results indicate that our pipeline not only accurately captures fungal signatures of PCWDEs, but also has a good discrimination power against the protein sequences from closely related enzymes to the PCWDEs. When comparing the average number of genes per species, plant genomes present the largest number (39.00 genes per genome), followed by Oomycetes (28.60) and fungi (20.01). Existence of signatures of fungal PCWDEs in other kingdoms suggests that these domains are quite universal and they could have diverse roles along with their niches and life styles.

Understandably, the most commonly found enzymes are related to the process of breaking the bond within dimer or polymer of glucose or mannose, as they are the most simple sugar sources that can be readily utilized by the life organisms [12]. The most common gene found in 304 genomes is alpha-glucosidase (Type 1), which hydrolyzes disaccharides and is usually involved in the endmost step of polysaccharide catabolism. In the second place, alpha-mannosidases (Type 1 and 2), cleaving alpha-form of mannose polymers, are found in at least 238 genomes (Additional file 1). The products of these two genes could be considered as PCWDEs, as they are involved in catabolism and turnover of plant *N*-glycans [13].

According to the identification results, fungi are the only taxon predicted to have genes encoding endoarabinase, alpha-glucuronidase, cutinase, endoxylanase (Type 2) and cellobiohydrolase (Type 2). In addition, three genes encoding pectin-degrading enzymes are found only in fungi and Oomycetes (pectin lyase, pectate lyase and rhamnogalacturonan lyase).

**Figure 2:**
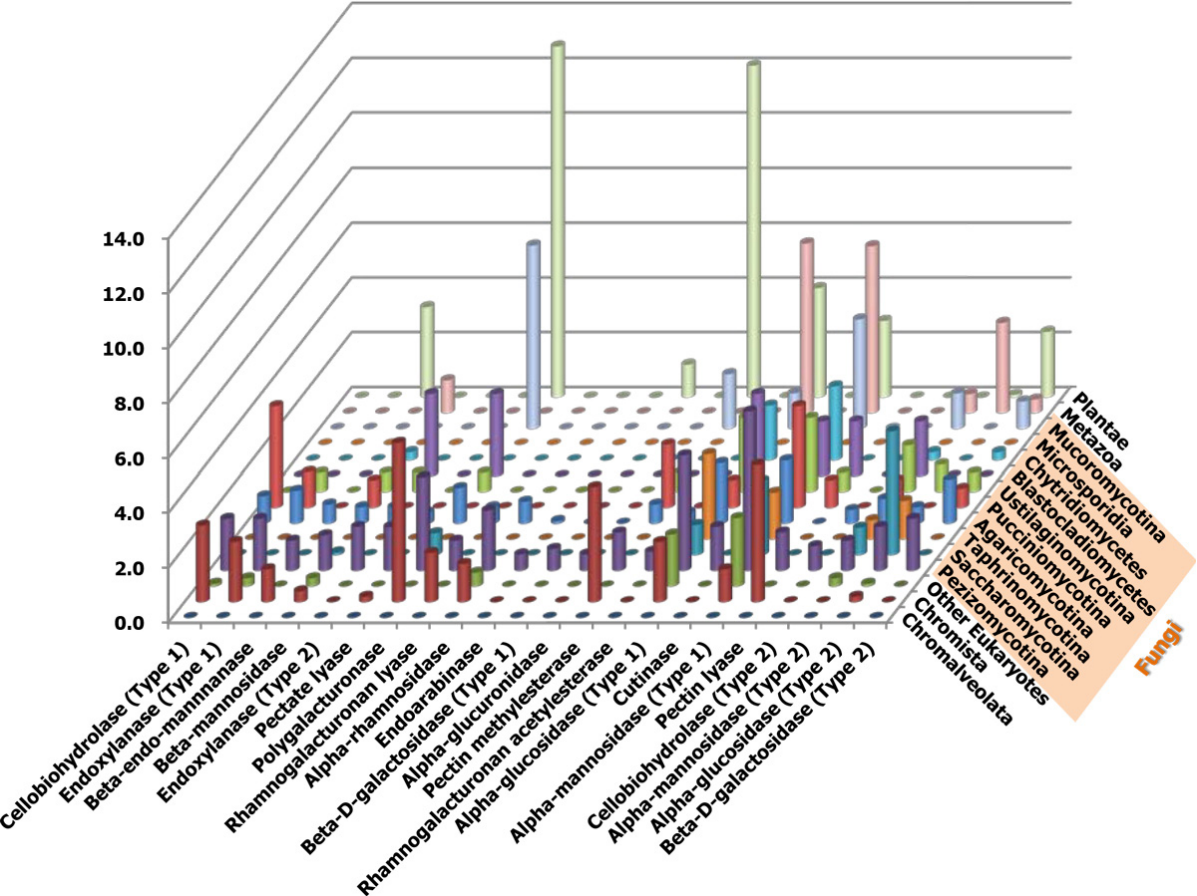
**Distribution of gene families over taxonomy**. The average numbers of predicted genes for each gene family are plotted against the Phylum-level of taxonomy. Non-fungal taxa are condensed for comparison with the numbers of fungal subphyla.

When considered parasitic life style of *Plasmodium *spp., it should come as no surprise that genes encoding PCWDEs are not predicted in these species, because they utilize molecular machineries from their hosts [14]. On the contrary, species from the Kingdoms Metazoa only have genes that are involved in basic polysaccharide degradation, such as mannosidases and glucosidases. In plants, two pectinolytic enzymes, PG and pectin methylesterase, are highly enriched that are essentially required for cell wall extension and fruit ripening [15]. In fungi and Oomycetes, however, more diverse gene families are found, especially in Pezizomycotina and Oomycetes. Among the species in Pezizomycotina, all of the 22 gene families are predicted, and PGs and pectate lyases are the most frequently found. Many enzymes which could be used as arsenal for invading plant cells are found only in fungi and Oomycetes, such as cutinase, endoxylanase (Type 2), pectate lyase and pectin lyase that imply their roles in pathogenesis (Figure 2). Secretome analysis by using Fungal Secretome Database (FSD; http://fsd.snu.ac.kr/) [16] has shown that 91.28% of these enzymes, on average, are predicted to be secretory (Table 2), indicating their importance at the apoplastic interface between fungal and host cell walls. Moreover, particularly in case of *M. oryzae*, 33 predicted PCWDEs are detected by either of *in planta *apoplastic secretome analysis or transcriptome profiling experiments [17,18]. These 33 PCWDEs also include three cutinases, eight endoxylanases, three pectate lyases and two PGs, suggesting their critical roles for successful infection to the host cells (Additional file 2).

**Table 2:** Secretory potential of PCWDEs in fungi and Oomycetes

	Number of Fungal/Oomycete Genes	ClassSP*	ClassSP^3^*	ClassSL*	Number of Secretory Proteins *
Cutinase	112	101	1	0	102 (91.07%)
Endoxylanase (Type 1)	168	152	3	0	155 (92.26%)
Endoxylanase (Type 2)	122	112	1	0	113 (92.62%)
Pectate lyase	119	108	2	0	110 (92.44%)
Pectin lyase	130	110	5	1	116 (89.23%)
Polygalacturonase	392	343	12	1	356 (90.82%)

* ClassSP, ClassSP^3 ^and ClassSL indicate the classes of secretory proteins defined in the FSD [16]. The number of secretory proteins is the sum of the three classes. Proportion of sequences with secretory potential is shown in parenthesis.

### Differential distribution of PCWDEs by life styles

A total of 215 fungal and Oomycete genomes are divided into five groups of life styles; animal pathogen, opportunistic animal pathogen, plant pathogen, parasite and saprophyte. *Tremella mesenterica*, a parasite of wood-decaying fungi in the genus *Peniophora*, is predicted to have accessary enzymes to break down di-/oligo-saccharides. Analogous composition of the genes is found in animal pathogens. They do not have the genes belonging to at least 15 gene families, only presenting genes encoding enzymes for polysaccharide degradation including alpha-glucosidase and alpha-/beta-mannosidase (Additional file 3). As their host range is limited to animals, it is natural that they do not encode pectin- or xylan-degrading enzymes.

The distribution of opportunistic animal pathogen could be divided into two subgroups, species in Pezizomycotina and Saccharomycotina. Among the opportunistic animal pathogen, most of PCWDEs are found in the species belonging to Pezizomycotina, while only alpha-/beta-mannosidase and alpha-glucosidase are found in three *Candida *spp. (Additional file 3) This result supports that duplication and loss events of genes encoding PCWDEs might be mainly driven by taxonomic divergence. Gene distribution in plant pathogens is quite diverse and much more genes are enriched in species belonging to Pezizomycotina. In the subphylum Pezizomycotina, pectate/pectin lyase and PG are intensively enriched enzymes that are known to be responsible for pathogenicity of fungal pathogens [5-7,19,20] (Additional file 3).

### Differential distribution of PCWDEs among plant-associated fungi

**Figure 3:**
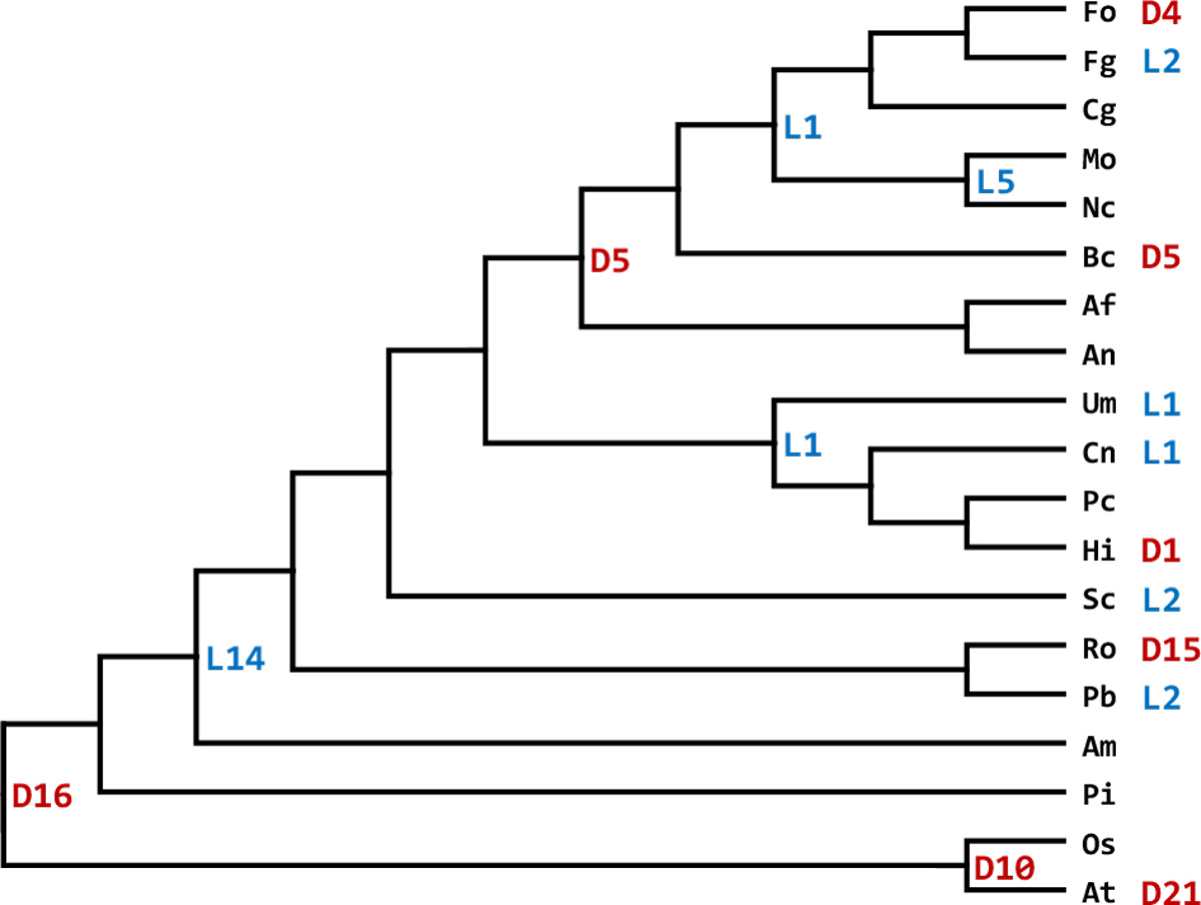
**Reconciled tree of PGs**. The reconciled tree for PGs from 34 species in FGGS. Genes encoding PG are only found in 19 species. The other species which do not have genes encoding PGs are not included in this figure. The numbers of duplication (D) and loss (L) events are shown in the corresponding internal nodes. The numbers of events at species-level are presented next to the name of leaf nodes. Species names are abbreviated as the followings: Fo (*Fusarium oxysporum*), Fg (*Fusarium graminearum*), Cg (*Colletotrichum graminicola *M1.001), Mo (*Magnaporthe oryzae *70-15), Nc (*Neurospora crassa*), Bc (*Botrytis cinerea*), Af (*Aspergillus fumigatus *Af293), An (*Aspergillus nidulans*), Um (*Ustilago maydis *521), Cn (*Cryptococcus neoformans *var. *grubii *H99), Pc (*Phanerochaete chrysosporium*), Hi (*Heterobasidion irregular *TC 32-1), Sc (*Saccharomyces cerevisiae *S288C), Ro (*Rhizopus oryzae*), Pb (*Phycomyces blakesleeanus*), Am (*Allomyces macrogynus*), Pi (*Phytophthora infestans*), Os (*Oryza sativa*) and At (*Arabidopsis thaliana*).

Wood-decaying fungi attack and digest moist wood, causing diverse rot diseases. Interestingly, rot fungi do not possess as many genes encoding PCWDEs as plant pathogens do. This is mainly because there is no duplication event after divergence of Ascomycota and Basidiomycota, except species-level events (Figure 3). In fact, unlike crop pathogenic fungi, ligninolytic enzymes, such as laccases and peroxidases, are more important in wood-decaying fungi that are essential to cause rot symptoms [21]. Five rot fungi included in this analysis are *Phanerochaete chrysosporium*, *Pleurotus ostreatus *PC9, *Dichomitus squalens*, *Heterobasidion irregulare *TC 32-1 and *Serpula lacrymans *which cause either brown rot, red rot, white rot or root rot, respectively. No pectin lyase-encoding gene is predicted from their genomes and only at most three copies of PG-encoding genes are predicted. In contrast, important plant pathogens such as *Phytophthora infestans*, *Colletotrichum higginsianum*, *Fusarium oxysporum *and two *Verticillium *spp. have at least 5 and 11 genes encoding pectin lyase and PG, respectively (Additional file 3). It supports that those highly enriched PCWDEs in plant pathogens are likely to be utilized within pathogenic interactions with a host, rather than decaying dead materials.

### Tracking evolutionary history of PGs

Among the pectin-degrading enzymes, PG is the most frequently found one. However, genes encoding PG are found only in Oomycetes, fungi and plants. This is might be due to the fact that PG is known to be involved in ripening of fruits for plants and rotting process especially by fungi [15]. For fungi, plant pathogens in particular, to successfully colonize on plant surface, they need to pass through the primary cell wall where pectin is highly concentrated [22]. Although some PGs are proven to be irrelevant with pathogenicity [23], majority of them would play roles outside fungal cells when considering that their target substrate is always outside fungal cell. In addition, 356 out of 392 putative PGs from fungi and Oomycetes are predicted to be secretory [16] (Table 2).

To investigate evolutionary track of a catalytic domain of PGs, genes from 34 species are selected (Table 3). As 15 species do not have the predicted genes, a gene tree and a species tree of the remaining 19 species are subjected to reconciliation analysis. Interestingly, the reconciled tree show intensive gene duplications and losses. In particular, losses only occurred in fungi, not in *Phytophthora infestans *and plants. All the fungi analysed have gone through at least 14 losses. The highest number of losses that had occurred is 20, where detected in *Neurospora crassa *and *M. oryzae *(Figure 3). The common ancestral gene(s) would have existed before the divergence of plants and fungi, and a large loss of PGs occurred at divergence between fungi and Oomycetes. After entering into fungi, another duplication event occurs at the divergence between the phyla Ascomycota and Basidiomycota. This duplication has preserved only in *Aspergillus *spp. and *B. cinerea*, while the other ascomycetes have undergone at least one loss event (Figure 3). These gain and loss events happened along with taxonomic hierarchy, rather than different fungal life styles. However, there have been duplication and loss events at species-level in 10 species, supporting that adaptation to local environments might partly contribute the evolution of the PGs. In accordance with the whole genome duplication and expansion of gene families in *Rhizopus oryzae *[24], a dramatic duplication event is detected at the degree of 15, presenting 18 predicted PGs (Figure 3).

**Table 3:** List of genomes for phylogenomic analysis

Species Name	Kingdom	Phylum	Subphylum	Life Style*
*Aspergillus fumigatus *Af293	Fungi	Ascomycota	Pezizomycotina	Animal pathogen
*Aspergillus nidulans*	Fungi	Ascomycota	Pezizomycotina	Saprotroph
*Blumeria graminis*	Fungi	Ascomycota	Pezizomycotina	Plant pathogen (Biotroph)
*Botrytis cinerea*	Fungi	Ascomycota	Pezizomycotina	Plant pathogen (Necrotroph)
*Coccidioides immitis *RS	Fungi	Ascomycota	Pezizomycotina	Animal pathogen
*Colletotrichum graminicola *M1.001	Fungi	Ascomycota	Pezizomycotina	Plant pathogen (Hemibiotroph)
*Fusarium graminearum*	Fungi	Ascomycota	Pezizomycotina	Plant pathogen (Necrotroph)
*Fusarium oxysporum*	Fungi	Ascomycota	Pezizomycotina	Plant pathogen (Necrotroph)
*Histoplasma capsulatum *H88	Fungi	Ascomycota	Pezizomycotina	Animal pathogen
*Magnaporthe oryzae *70-15	Fungi	Ascomycota	Pezizomycotina	Plant pathogen (Hemibiotroph)
*Mycosphaerella graminicola*	Fungi	Ascomycota	Pezizomycotina	Plant pathogen (Hemibiotroph)
*Neurospora crassa*	Fungi	Ascomycota	Pezizomycotina	Saprotroph
*Podospora anserine*	Fungi	Ascomycota	Pezizomycotina	Saprotroph
*Candida albicans*	Fungi	Ascomycota	Saccharomycotina	Animal pathogen
*Saccharomyces cerevisiae *S288C	Fungi	Ascomycota	Saccharomycotina	Saprotroph
*Schizosaccharomyces pombe*	Fungi	Ascomycota	Taphrinomycotina	Saprotroph
*Heterobasidion irregular *TC 32-1	Fungi	Basidiomycota	Agaricomycotina	Plant pathogen (Necrotroph)
*Laccaria bicolor*	Fungi	Basidiomycota	Agaricomycotina	Saprotroph
*Phanerochaete chrysosporium*	Fungi	Basidiomycota	Agaricomycotina	Saprotroph
*Serpula lacrymans*	Fungi	Basidiomycota	Agaricomycotina	Saprotroph
*Cryptococcus neoformans *var. *grubii *H99	Fungi	Basidiomycota	Agricomycotina	Animal pathogen
*Melampsora laricis-populina*	Fungi	Basidiomycota	Pucciniomycotina	Plant pathogen (Biotroph)
*Puccinia graminis*	Fungi	Basidiomycota	Pucciniomycotina	Plant pathogen (Biotroph)
*Ustilago maydis *521	Fungi	Basidiomycota	Ustilaginomycotina	Plant pathogen (Hemibiotroph)
*Allomyces macrogynus*	Fungi	Blastocladiomycota	N/D	Saprotroph
*Batrachochytrium dendrobatidis *JAM81	Fungi	Chytridiomycota	N/D	Animal pathogen
*Phycomyces blakesleeanus*	Fungi	Zygomycota	Mucoromycotina	Saprotroph
*Rhizopus oryzae*	Fungi	Zygomycota	Mucoromycotina	Saprotroph
*Phytophthora infestans*	Chromista	Oomycota	Oomycotina	Plant pathogen
*Arabidopsis thaliana*	Viridiplantae	Streptophyta	N/D	
*Oryza sativa*	Viridiplantae	Streptophyta	N/D	
*Dorosophila melanogaster*	Metazoa	Arthropoda	N/D	
*Caenorhabditis elegans*	Metazoa	Nematoda	N/D	
*Homo sapiens*	Metazoa	Chordata	Craniata	

* Information about life style and host ranges are shown only for 29 fungal and Oomycete species.

## Utility

### Web interfaces

**Figure 4:**
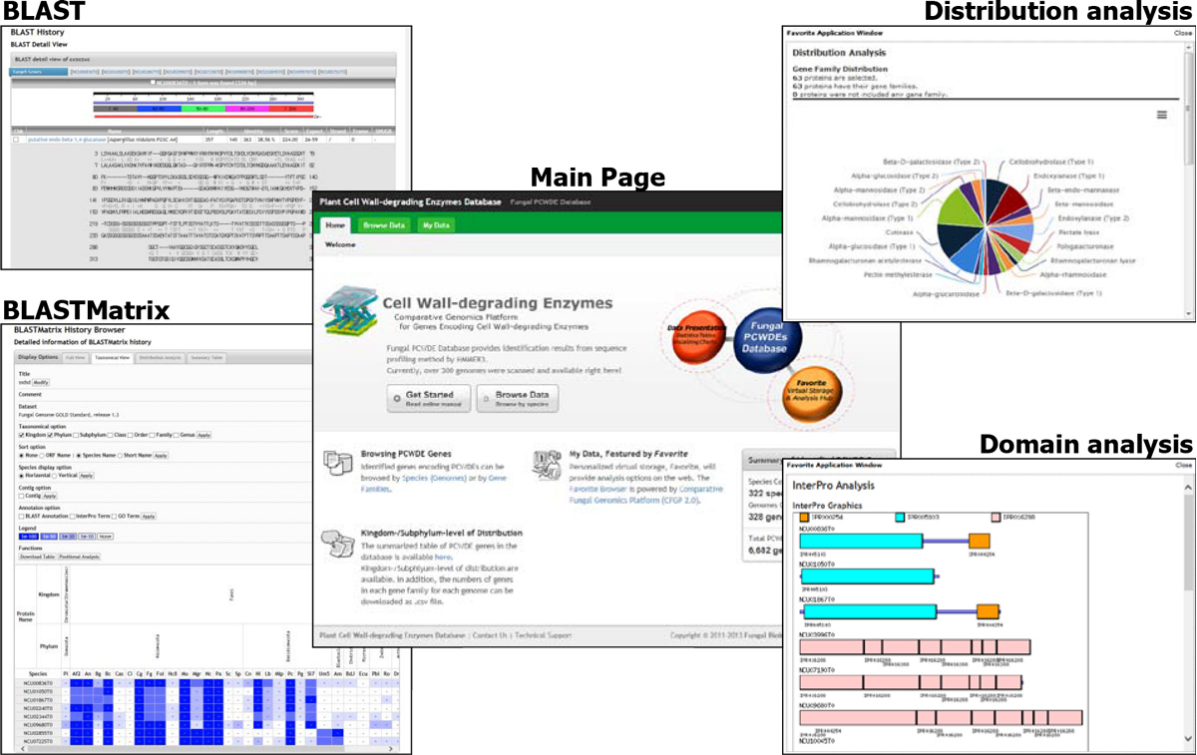
**Web utility of FPDB**. The FPDB supports BLAST with user provided sequences or sequences in a Favorite. BLASTMatrix is also available for sequences in a Favorite, providing distribution of homologous genes throughout a data set selected. In Favorite Browser, distribution of gene families and protein domains can be browsed.

To provide user-friendly and intuitive user experience, the web pages of the FPDB are concisely designed by adopting Data-driven User Interface of Comparative Fungal Genomics Platform (CFGP 2.0; http://cfgp.snu.ac.kr/) [25]. *In silico *identified genes encoding PCWDEs can be browsed by either species or gene families. In the Species Browser, kingdom-level and phylum-level of statistics are provided as well as download option for distribution of PCWDEs in all the 328 genomes. In the Gene Family Browser, distribution along with subphylum-level taxonomy is available for every gene family, providing a glimpse of distribution across the large number of genomes (Figure 4).

### Cross-link with the CFGP 2.0 for further analysis

The FPDB web site supports "Favorite", a personal storage and analysis hub powered by the CFGP 2.0 [25]. In the My Data menu, users can create and manage their own data collections, which are synchronized with the CFGP 2.0. The FPDB website is also featured with i) gene family distribution, ii) BLAST search, iii) BLASTMatrix search and iv) functional domain browser. Users can also use their Favorites in the CFGP 2.0, providing more analysis options.

## Conclusions

The FPDB is developed to take the advantages of a number of fully sequenced fungal genomes and to provide fungi-centric platform for studying PCWDEs. The FPDB could be used for i) selection of target genes that affect fungal pathogenicity, ii) making *in silico *combinations of PCWDEs for degrading certain substrate and iii) starting material for fungal evolutionary studies of gene families belong to PCWDEs. The web resource we developed provides i) kingdom-/subphylum-wide overview of PCWDEs in fungi with browsing pages and distribution charts, ii) domain visualization function, iii) homology search functions (BLAST and BLASTMatrix) and iv) a bridge to connect with the CFGP 2.0 for flexible data exchange and further analysis. To provide more comprehensive research environment, the FPDB will be updated with more PCWDE gene families, useful analysis tools and up-to-date genome sequences. Taken together, the FPDB can serve as a fungi-centric comparative genomics resource for studying PCWDEs.

## Methods

### Collection of protein sequences for construction of sequence profiles

155,095 protein sequences covering 33 gene families were downloaded from NCBI Protein Database with keywords of gene family names. To investigate fungi-centered gene distribution and ensure representativeness of sequence profiles, sequences that are partial or from other kingdoms were discarded, hence 1,344 fungal protein sequences were chosen for building 22 sequence profiles, including five subfamilies (Table 1). In particular, the sequence profile for beta-D-galactosidase (Type 2) was constructed by the protein sequences collected from the UniProtKB/SwissProt [11].

### Collection of proteome sequences

Protein sequences of 328 genomes (Additional file 1) were obtained from the standardized genome warehouse of the CFGP 2.0 [25].

### A constructed pipeline for genes encoding PCWDEs

To identify genes encoding PCWDEs, HMMER3 package [26] was exploited to build sequence profiles and predict putative genes. InterPro scan [27] was also used in determination of consensus domains for each gene family. If there is more than one domain profile for one gene family, they were divided into subfamilies with a designation like "Type 1" and "Type 2". Concatenated domain sequences for each gene family were subjected to multiple sequence alignment by using MUSCLE built in MEGA5 [28]. Subsequently, the alignments were manually trimmed, then used as input when building sequence profiles by using *hmmbuild*. *hmmsearch *in the HMMER3 package [26] was used for identifying candidate genes encoding PCWDEs from the 328 proteomes from 322 species (Figure 1 and Table 1).

### Elimination of redundancy

Because certain gene families could share high sequence homology, one gene could be predicted in multiple gene families. To eliminate this redundancy, the gene family which marked the highest score was assigned and the rest of predictions for that sequence were discarded.

### Evaluation of the pipeline

In order to evaluate the confidence level of the pipeline, we prepared positive and negative sets from UniProtKB/SwissProt [11]. The positive set was defined as the protein sequences annotated as the PCWDEs investigated in this study. Subsequently, the protein sequences used in construction of the 22 sequence profiles were filtered out from the positive set. The protein sequences of enzymes that are closely related to PCWDEs were determined as the negative set. Only the fungal sequences having UniProt accession were retrieved among the sequences of glycosyltransferase (GT), polysaccharide lyase (PL) and carbohydrate esterase (CE) from the CAZY database [8]. GT, PL and CE are carbohydrate active enzymes like PCWDEs, but they have different catalytic activities. Therefore it makes these sequences a good negative data set to evaluate the discrimination power of the PCWDE identification pipeline. In total of 128 and 344 sequences were selected for the positive and negative sets, respectively.

### Reconciliation analysis

A phylogeny of genomes was constructed by CVtree2 [29]. Whole proteome sequences were used as input of the CVtree2 with K-tuple length of seven. Distance matrix was converted into neighbor-joining tree by *neighbor *in PHYLIP package v3.69 [30]. Multiple sequence alignment and construction of phylogenetic tree were performed by using T-Coffee [31] and MEGA5 [28], respectively. To investigate gene duplications and losses during the evolution, reconciliation analysis was performed by using Notung 2.6 [32]. For phylogenomic analyses, genomes and proteomes were prepared from 34 species covering 28 fungi, one Oomycete, two plants and three animals. The 28 fungi cover 6 phyla with diverse life styles and infection styles (Table 2).

## Availability of supporting data

All data described in this paper can be freely accessed through the FPDB web site at http://pcwde.riceblast.snu.ac.kr/ via the latest versions of Google Chrome, Mozilla Firefox, Microsoft Internet Explorer (9 or higher) and Apple Safari. The data sets supporting the results of this article are included within the article and its additional files.

## Competing interests

The authors declare that they have no competing interests.

## Authors' contributions

JC and YHL designed this project. JC developed the database, web interfaces and identification pipeline. JC and KTK conducted data analysis. JC, KTK, JJ and YHL wrote the manuscript. All the authors read and approved the final manuscript.

## Supplementary Material

Additional file 1 **Summary of the number of predicted genes encoding PCWDEs in 328 genomes**. List of taxonomically ordered 328 genomes archived in the FPDB. The number of predicted genes for each gene family is listed. Click here for file

Additional file 2 **Expression of PCWDEs in *M. oryzae *reported in the previous studies**. The 33 genes encoding PCWDEs in *M. oryzae *that are expressed *in planta *apoplastic secretome analysis and/or transcriptomic profiling are listed. Click here for file

Additional file 3 **Distribution of genes encoding PCWDEs in 215 fungal or Oomycete genomes**. The numbers of genes for each gene family are listed along with the list of 215 fungi and Oomycetes which is ordered by life style and taxonomy. Click here for file

## Declarations

The publication costs for this article were funded by the National Research Foundation of Korea grant (2008-0061897).

This article has been published as part of *BMC Genomics *Volume 14 Supplement 5, 2013: Twelfth International Conference on Bioinformatics (InCoB2013): Computational biology. The full contents of the supplement are available online at http://www.biomedcentral.com/bmcgenomics/supplements/14/S5.
